# Antibacterial effects of coniferyl alcohol-derived dehydrogenation polymer on chlamydial infection *in vitro*

**DOI:** 10.3389/fchem.2025.1654478

**Published:** 2025-10-10

**Authors:** Anna Pfundner, Tamara Weinmayer, Nora Geissler, Ana Kovacevic, Dragica Spasojevic, Ksenija Radotic, Marijana Stojanovic, Irma Schabussova, Ursula Wiedermann, Aleksandra Inic-Kanada

**Affiliations:** 1 Institute of Specific Prophylaxis and Tropical Medicine, Center for Pathophysiology, Infectiology and Immunology, Medical University of Vienna, Vienna, Austria; 2 Institute of Immunology, Virology, Vaccines and Sera – Torlak, Belgrade, Serbia; 3 Institute for Multidisciplinary Research, University of Belgrade, Belgrade, Serbia; 4 Institute for Biological Research “Siniša Stanković” - National Institute of Republic of Serbia, University of Belgrade, Belgrade, Serbia

**Keywords:** chlamydial infection, sexually transmitted infection, *Chlamydia trachomatis*, treatment, lignin, dehydrogenation polymer

## Abstract

**Background:**

*Chlamydia trachomatis*, Gram-negative obligate intracellular bacteria, are a leading cause of sexually transmitted diseases worldwide, often causing severe complications. With no vaccine available and concerns about potential antibiotic resistance, the need for novel treatments is urgent. Dehydrogenation polymer of coniferyl alcohol in alginate hydrogel (DHP/Alg) has not yet been tested against chlamydial infections.

**Material and Methods:**

The cytotoxicity of DHP/Alg on A2EN genital epithelial cells was assessed by measuring cell viability. To investigate its effects on *Chlamydia*-infected cells, we employed flow cytometry-based assays, fluorescence microscopy, and quantitative PCR (qPCR). Additionally, adhesion assays were performed to examine whether DHP/Alg interferes with *Chlamydia* entry into host cells.

**Results:**

No cytotoxic effects of DHP/Alg in tested concentrations on A2EN cells were observed, confirming its safety. Infection and adhesion assays demonstrated a significant reduction in infection levels, suggesting that DHP/Alg directly targets *Chlamydia* elementary bodies, thereby disrupting their ability to adhere and initiate infection. Fluorescence microscopy revealed that 75 μg/mL DHP/Alg is the most effective dose evaluated to reduce chlamydial infection *in vitro*, as indicated by the decreased number of inclusions. These findings were further confirmed by qPCR analysis.

**Conclusion:**

Our results suggest that DHP/Alg is a promising therapeutic option against chlamydial infections. The significant reduction in adhesion levels indicates that DHP/Alg effectively interferes with the initial stages of infection.

## 1 Introduction

*Chlamydia trachomatis* (Ct) is a leading cause of sexually transmitted infections (STIs) and the etiological agent of trachoma, the world’s most common infectious cause of blindness (de la Maza et al., 2021; Hu et al., 2013). According to the World Health Organization, there were an estimated 129 million new cases of genital chlamydial infection in 2020, making it one of the most prevalent bacterial STIs globally (WHO, 2025).

Genital Ct infections can lead to severe reproductive health complications, including pelvic inflammatory disease (PID), infertility, ectopic pregnancies, and chronic pelvic pain (Sharma and Khan, 2025). The economic burden is substantial: in the United States alone, the annual direct medical costs of *Chlamydia* and its complications exceed US $500 million, with total lifetime costs for all incident STIs (excluding HIV) reaching US $2.4 billion; women bear the majority of these costs due to the high rate of complication-related healthcare use and productivity losses (Chesson et al., 2021).

Despite decades of research, vaccine development for Ct has faced significant challenges, including the complexity of eliciting long-lasting mucosal immunity, antigenic variation among serovars, and incomplete protection observed in preclinical and early clinical trials (de la Maza et al., 2021; Poston et al., 2025; de la Maza et al., 2023; de la Maza et al., 2020; de la Maza et al., 2017; Boje et al., 2016; I et al., 2016; Inic-Kanada et al., 2015; Pollock et al., 2024). Given its public health burden and the lack of an available vaccine (de la Maza et al., 2020), effective control measures and prophylactic strategies are urgently needed (Elwell et al., 2016; Murray and McKay, 2021).

Although antibiotic therapy, especially with azithromycin, is effective for treating Ct infections, its impact is limited to individuals who actively seek medical care (Murray and McKay, 2021). A significant challenge is that many Ct infections remain asymptomatic (Detels et al., 2011), making regular STIs screening essential for effective disease management (Murray and McKay, 2021). While antibiotics are highly effective in many cases, there is a concern that Ct infections may persist despite treatment, potentially leading to complications like PID and long-term reproductive health issues.

In addition to targeted treatment of active infections, prophylactic antibiotic strategies such as doxycycline post-exposure prophylaxis (DoxyPEP) have been proposed for reducing STI incidence. While potentially beneficial, the widespread use of DoxyPEP, which involves lower doses and shorter durations than treatment of active infection, may contribute to the emergence of antibiotic resistance over time. Historical evidence from livestock production shows that prophylactic tetracycline use in swine facilitated the spread of tetracycline-resistant bacterial strains in Europe (Van Boeckel et al., 2015), and tetracycline-resistant *Chlamydia suis* has been generated and observed in both *in vitro* and *in vivo* studies (Borel et al., 2012; Marti et al., 2021; Unterweger et al., 2020). Consequently, any implementation of prophylactic antibiotic measures should be accompanied by robust resistance surveillance (Bachmann et al., 2024).

These challenges underscore the need for novel therapeutic alternatives to mitigate the risks of treatment failure and reduce reliance on antibiotics.

Natural products have emerged as promising candidates for anti-*Chlamydia* therapeutics (Brown et al., 2016; Hou et al., 2022). Carrageenans, polysaccharides derived from seaweed, have been shown to reduce Ct infectivity (Inic-Kanada et al., 2018). Polyphenolic compounds, known for their antioxidant and antimicrobial properties, have also demonstrated anti-*Chlamydia* potential (Yamazaki et al., 2003; Yamazaki et al., 2005), along with lipid-based (Bergsson et al., 1998) and proteinaceous compounds, such as antimicrobial peptides (Ballweber et al., 2002). Also, probiotics like *Lactobacillus* spp (Mastromarino et al., 2014) and polyherbal formulations have exhibited notable anti-chlamydial properties (Talwar et al., 2000). These findings highlight the potential of natural products and formulations as alternative or complementary strategies to conventional antibiotics.

Lignin, the second most abundant polymer on Earth after cellulose, is a complex aromatic biopolymer with known antimicrobial properties (Lobo et al., 2021; Spasojević et al., 2016). However, the heterogeneity of natural lignin, resulting from the diverse sources and extraction methods, poses challenges for biomedical applications (Sugiarto et al., 2022). To overcome this, synthetic lignin models, such as dehydrogenation polymer of coniferyl alcohol (DHP), provide a more uniform and controllable framework for research and development.

Recent studies have demonstrated that low-molecular weight (LMW) DHP fractions embedded in a cellulose matrix exhibit antimicrobial activity against a range of bacterial strains (Zmejkoski et al., 2018). DHP incorporated into alginate hydrogel (DHP/Alg) has shown antimicrobial effects against both Gram-positive and Gram-negative bacteria, while exhibiting no toxicity toward human epithelial cell lines HCjE and HepG2 (Spasojević et al., 2016). Furthermore, LMW lignin fractions (2–3 kDa), suspended in alginate, have been found to promote wound healing by reducing inflammation and infection while providing a soothing effect on the skin. These fractions have also exhibited antimicrobial activity against *Staphylococcus* spp. (Spasojević et al., 2024).

Alginate, a naturally occurring polysaccharide derived from algae, forms hydrogels through ionic crosslinking. It is widely recognized for its biocompatibility, accessibility, and cost-effectiveness, making it an attractive material for drug delivery and wound healing application (Pires et al., 2024; Zhang et al., 2021). As a hydrogel matrix, alginate ensures prolonged exposure to the active DHP component on the site of application (Zhang et al., 2021).

Given DHP’s demonstrated antibacterial properties, we hypothesize that it may also exhibit activity against Ct infections. Although Ct is an obligate intracellular pathogen, its unique infection mechanisms suggest that lignin-based compounds could interfere with its adhesion or early infection-stage process. Investigating DHP’s potential against Ct could offer a novel therapeutic approach for intracellular bacterial infections.

## 2 Materials and methods

### 2.1 Chlamydial strains

Ct serovar E (CtE) was propagated in McCoy cells, harvested by mechanical disruption using glass beads, and purified via ultracentrifugation at 160,000 × g using a discontinuous Gastrografin (Bayer, Germany) gradient (40/44/54%). Purified elementary bodies (EBs) were collected from the 40/44% interface, washed, and stored in Sucrose-Phosphate-Glutamic acid (SPG) buffer for further use.

### 2.2 Labeling of EBs

EBs of CtE were labelled with carboxyfluorescein succinimidyl ester (CFSE) (eBisocience, 65-0850, Invitrogen, United States) as described by Schnitger et al. (2007). Briefly, EBs were resuspended in SPG buffer and incubated with 20 µM CFSE for 90 min at room temperature (RT) in the dark. Labelled EBs were pelleted (18,000 x g in a high-speed centrifuge for 10 min) and excess dye was removed by three washes with 1% BSA in Dulbecco’s Phosphate-Buffered Saline (DPBS), each followed by centrifugation (18,000 x g, 10 min). Due to wash-associated losses, the final titer was estimated at 70% of the starting concentration. CFSE-labelled EBs were then used to infect A2EN cells.

### 2.3 Cell culture

Immortalized human endocervical epithelial A2EN cells were cultured in Keratinocyte Serum-Free Medium (KSFM, 10725-018, Gibco, Thermo Fisher Scientific, United States) supplemented with 12.5 mg of supplied bovine pituitary extract, 0.2 ng/mL EGF (Supplements for KSFM, 37000-015, Gibco, Thermo Fisher Scientific, United States), 0.4 mM CaCl_2_ and 1% penicillin/streptomycin (P06-07100, Pan Biotech, Germany) at 37 °C/5% CO_2_ and 95% humidity in standard cell culture flasks (90026 and 90076, TPP, Switzerland). Cells were passaged at 70%–80% confluency. For passaging, cells were detached using 0.05% Trypsin/0.02% EDTA (P10-023100, Pan Biotech, Germany) and neutralized with an equal volume of Neutralization medium (DMEM/Hams F12 (SH30023.01, Cytiva, US) with 10% FCS (9665, Sigma, US) and 1% penicillin/streptomycin. Cells were then centrifuged for 4 min at 2,600 × g at RT, the pellet was resuspended in KSFM and transferred to a new flask.

### 2.4 DHP/alg stock preparation

The DHP was synthesized following the method outlined by Radotić et al. (1994). The obtained compound was air-dried. The DHP mixture consisted of 65.1% molecules with a molecular weight >10 kDa, 19.2% between 10 and 3 kDa, 11% between 3 and 1 kDa, and <4.7% 1 kDa (Spasojević et al., 2023). For the stock solution, 10 mg DHP and 20 mg Alg (Sigma-Aldrich, United States) were solubilized in 50 µL 100% DMSO to ensure complete dissolution of DHP. This was then filled with 950 µL dH_2_O to reach a 10 mg/mL stock solution of DHP/Alg. This was diluted to 500 μg/mL starting solution in KSFM (without penicillin/streptomycin) prior to use, which was sterile filtered and further diluted to desired concentrations in the same medium. The concentrations mentioned always correspond to the DHP concentration, and the ratio of DHP:Alg is 1:2.

### 2.5 Cytotoxicity assay

A2EN cells were seeded at a density of 5,000 cells/well in a 96-well plate. The following day, medium was removed, and cells were treated with DHP/Alg (1–100 μg/mL for DHP in KSFM). Controls included medium-only (no cells), non-treated cells control, and 1 μg/mL azithromycin (treatment control). As an additional control, heat-inactivated EBs (processed in parallel) were included to test viability-independent effects; and the absence of inclusions on passage confirmed loss of infectivity. Here, cells were treated with DHP/Alg (75 and 100 μg/mL with respect to DHP in KSFM). Controls included medium-only (A2EN + medium), non-treated cells control (A2EN + heat-inactivated CtE), and 1 μg/mL azithromycin (treatment control, A2EN + heat-inactivated CtE + 1 μg/mL azithromycin).

After 48h, 10 µL of the Cell Counting Kit-8 (CCK-8, 96992, Sigma-Aldrich, US) reagent was added, and the plate was incubated for 4 h at 37 °C/5% CO_2_ and 95% humidity. Cell viability was measured at 450 nm using a microplate reader (Varioskan Flash, Thermo Scientific).

### 2.6 Infection assay via flow cytometry

Flow cytometric detection of A2EN cells infected with CFSE-labeled CtE EBs was performed in line with established protocols (Grasse et al., 2018; Schnitger et al., 2007). We used this assay as a rapid and cost-efficient pre-screen to compare four experimental conditions and to identify potential mechanistic differences. This approach allowed us to prioritize conditions before moving to the gold-standard cell culture method, which we then used to confirm quantification under the treatment-relevant condition.

**FIGURE 1:**
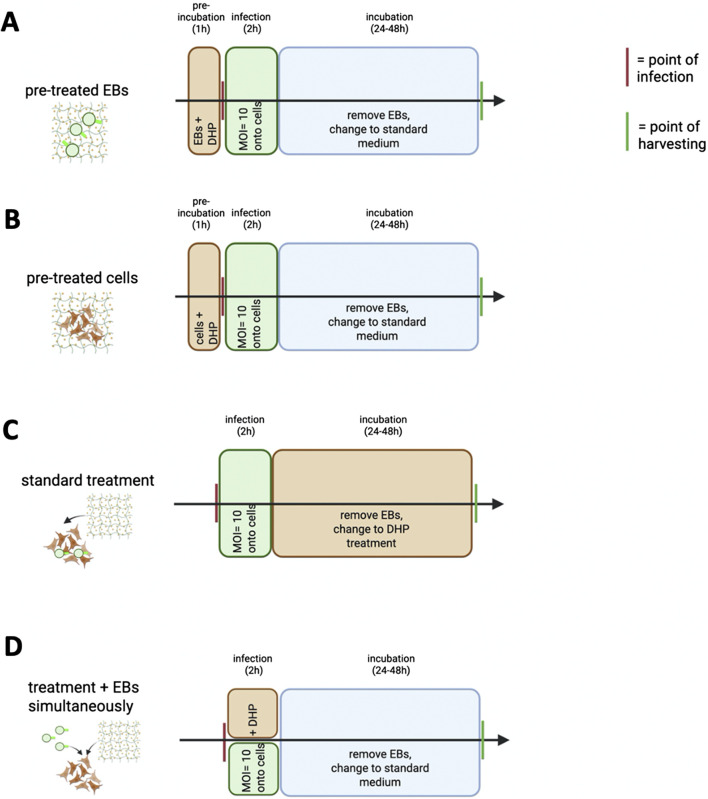
Graphical workflow summarizing the four treatment conditions used in infection assays. Infection was conducted with CtE EBs (MOI = 10). Each lane represents a distinct experimental setup: **(A)** Pre-incubation of EBs with DHP/Alg prior to infection. **(B)** Pre-treatment of cell monolayers with DHP/Alg prior to infection. **(C)** Treatment with DHP/Alg after infection. **(D)** Simultaneous addition of EBs and DHP/Alg to cell monolayers. Created with BioRender. When ‘DHP’ is mentioned, it refers to DHP/Alg.

A2EN cells were seeded at a density of 150,000 cells per well in a 24-well plate. The following day, cells were infected with CtE with a multiplicity of infection (MOI) of 10 and different treatment options were performed (Figure 1). DHP/Alg concentrations used were between 25–100 μg/mL with respect to DHP and were diluted to the needed concentration in KSFM without 1% penicillin/streptomycin. Controls included non-infected cells (cells incubated with medium alone) and infected cells without any treatment (cells were infected with labeled EBs).

#### 2.6.1 Pre-incubation of EBs with DHP/Alg prior to infection – “CtE pre-treated”

The labeled EBs were incubated with DHP/Alg in different concentrations for 1 h. These EBs were then used to infect A2EN cell monolayers. The plates were centrifuged at 900 *g* for 0.5 h at RT after infection to facilitate adhesion, which was followed by a 1.5 h incubation period at 37 °C/5% CO_2_ and 95% humidity. The medium was afterwards replaced by KSFM without 1% penicillin/streptomycin, and plates were incubated for 48 h at 37 °C/5% CO_2_ and 95% humidity before harvesting.

#### 2.6.2 Pre-treatment of cell monolayers with DHP/Alg prior to infection – “A2EN pre-treated”

DHP/Alg treatment was applied in different concentrations on the A2EN cell monolayers for 1 h and the medium was removed. The EBs were then added onto the cells and the plates were centrifuged at 900 *g* for 0.5 h at RT after infection to facilitate adhesion, which was followed by a 1.5 h incubation period at 37 °C/5% CO_2_ and 95% humidity. The medium was afterwards replaced by KSFM without 1% penicillin/streptomycin, and plates were incubated for 48 h at 37 °C/5% CO_2_ and 95% humidity before harvesting.

#### 2.6.3 Treatment with DHP/Alg after infection – “standard treatment”

Labeled EBs were used to infect A2EN cell monolayers and the plates were centrifuged at 900 *g* for 0.5 h at RT after infection to facilitate adhesion, which was followed by a 1.5 h incubation period at 37 °C/5% CO_2_ and 95% humidity. The medium was removed and replaced by DHP/Alg treatment in different concentrations. Plates were incubated for 48 h at 37 °C/5% CO_2_ and 95% humidity before harvesting.

#### 2.6.4 Simultaneous addition of EBs and DHP/Alg to cell monolayers – “treatment + EBs, simultaneously”

Labeled EBs and DHP/Alg treatment were simultaneously added on the A2EN cell monolayers and the plates were centrifuged at 900 *g* for 0.5 h at RT after infection to facilitate adhesion, which was followed by a 1.5 h incubation period at 37 °C/5% CO_2_ and 95% humidity. The medium was afterwards replaced by KSFM without 1% penicillin/streptomycin, and plates were incubated for 48 h at 37 °C/5% CO_2_ and 95% humidity before harvesting.

For harvesting, the medium was first removed and cells were washed with PBS. Trypsin (200 µL) was added per well to harvest the cells. As soon as cells were detached, an equal volume of Neutralization medium was added. The cell suspension was transferred to a 96-well plate in two steps: 200 µL was added first, followed by centrifugation at 2,600 × g for 4 min, then the remaining 200 µL was layered on top. Medium was then removed and cells were washed with PBS (centrifuged for 4 min at 2,600 x g). Then, the cells were fixed in 2% paraformaldehyde for 15 min at RT and washed again with PBS (centrifuged for 4 min at 2,600 x g). Pellets were resuspended in 150–180 µL 1% BSA in DPBS and flow cytometry analysis was performed on a Cytek Northern Lights cytometer. A minimum of 50,000 events per well were recorded.

The gating strategy included: (i) forward scatter (FSC) and side scatter (SSC) gating to exclude debris, (ii) FSC-A vs. FSC-H gating to select singlets, and (iii) CFSE fluorescence gating to identify infected cells. Quantification was based on the percentage of CFSE-positive cells in the singlet population. Although chlamydial inclusion size can vary under different treatments, the CFSE-based detection identifies infected cells regardless of inclusion morphology or size, ensuring comparability across conditions.

To complement these analyses, a Live/Dead staining was performed using the Zombie Red Fixable Viability Kit (BioLegend, 423109). The dye was diluted 1:2000 in PBS, and 100 µL were added to each well, followed by 20 min incubation at RT in the dark. Staining was stopped by adding 100 µL of 1% BSA/DPBS, and cells were washed with PBS. This step was performed immediately before fixation. The gating strategy for both the CFSE infection assay and Live/Dead staining, as well as the corresponding flow cytometry data, is provided as Supplementary Figures 2A-D.

### 2.7 Invasion assay

**FIGURE 2:**
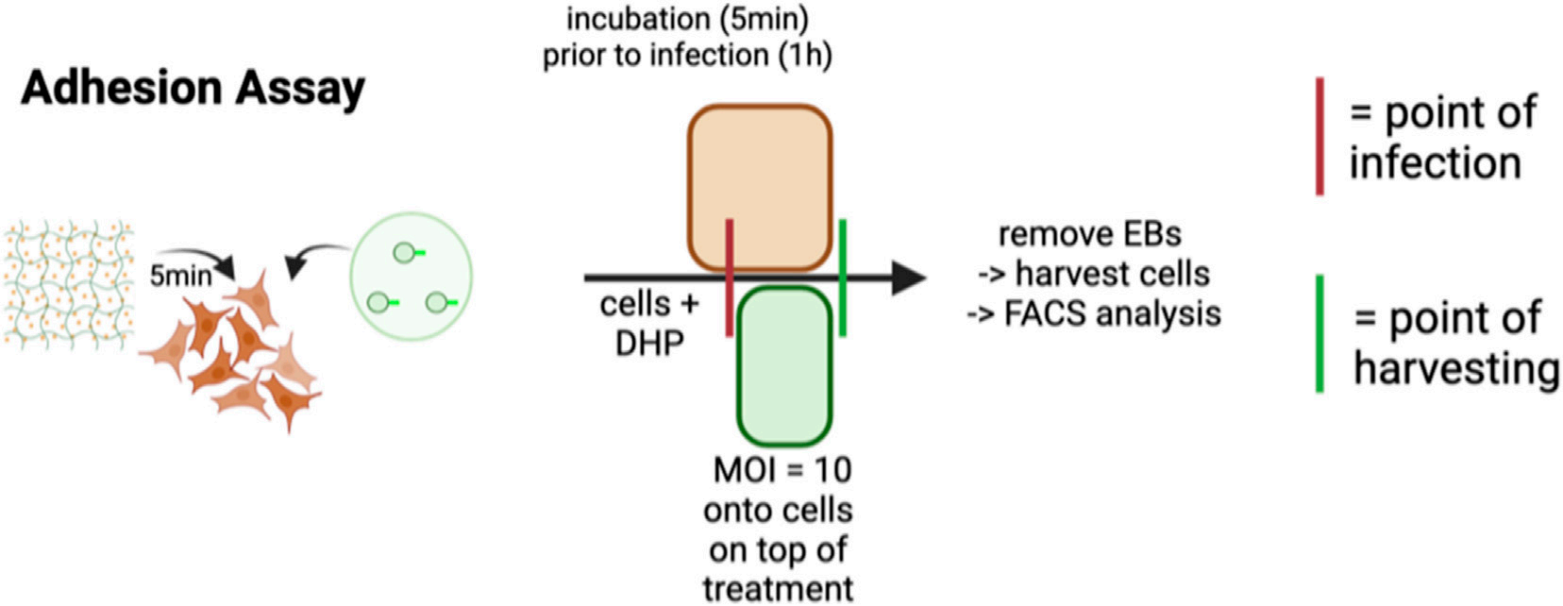
Graphical workflow of the adhesion assay via flow cytometry. Infection was conducted with CtE EBs (MOI = 10). When ‘DHP’ is mentioned, it refers to DHP/Alg. Created with BioRender.

A2EN cells were seeded at a density of 150,000 cells per well in a 24-well plate and grown overnight at 37 °C/5% CO_2_ and 95% humidity (Figure 2). Cells were pretreated with DHP/Alg (25–100 μg/mL for DHP) for 5 min prior to infection with CtE at an MOI of 10 for 1 h (at 37 °C/5% CO_2_ and 95% humidity) without centrifugation and then harvested. A high inoculum (MOI 10) was chosen to maximize initial infection and create a stringent condition to assess whether DHP can counteract invasion. Controls, fixation, and analysis followed Section 2.6.

### 2.8 Infection assay via fluorescence microscopy

A2EN cells were seeded at 50,000 cells per well on coverslips (diameter of 12 mm) in a 24-well plate. The next day, cells were infected with CtE (MOI of 1), centrifuged for 1 h to facilitate infection, and incubated for 1 h at 37 °C/5% CO_2_ and 95% humidity. Then, the medium was removed and replaced with DHP/Alg treatment at varying concentrations in KSFM (without penicillin/streptomycin). As a positive control of a working treatment 1 μg/mL azithromycin diluted in KSFM was used. As a negative control, instead of adding DHP/Alg treatment, only KSFM was used. Non-infected cells served as an additional control.

After 48 h, the medium was removed, and cells were washed with DPBS for 5 min and fixed with ice-cold methanol for 10 min at −20 °C, following another washing step. Coverslips were stained with 200 µL FITC-labelled anti-Chlamydia-LPS antibody (MA1-7339, Invitrogen, US) 1:20 diluted in 5% BSA in DPBS for 30 min at RT in the dark. The stain was removed, and cells were washed 3 × 5 min with DPBS and counterstained with 1 μg/mL 4′,6-diamidino-2-phenylindole (DAPI, D9542, Invitrogen, US). Coverslips were mounted on microscopic slides using DAKO fluorescent mounting medium and stored overnight at 4 °C. They were analyzed using the TissueFaxs microscope (TissueGnostics, Austria). Images were captured using a ×63 oil immersion objective, and inclusions were counted in 20 fields for semiquantitative measurement.

### 2.9 RT-qPCR

To quantify chlamydial load, the levels of 16S and 23S rRNA were measured using qPCR. A2EN cells were grown in T75 cell culture flasks until reaching approximately 1–1.5 × 10^6^ cells per flask. Cells were infected at an MOI of 1. One flask was left uninfected as a negative control. The flasks were centrifuged to facilitate infection and incubated for an additional hour at 37 °C/5% CO_2_ and 95% humidity. The Ct suspension was removed, and KSFM without 1% penicillin/streptomycin was added to the control group. For treatment groups, DHP/Alg at varying concentrations diluted in the same medium was added alongside an antibiotic control (1 μg/mL azithromycin). The flasks were incubated for 40 h at 37 °C/5% CO_2_ and 95% humidity.

After incubation, CtE was harvested via glass bead disruption. Cell debris was pelleted and the supernatant was centrifuged at 18,000 × g in high-speed centrifuge tubes, after which the pellet containing the CtE was resuspended in TE buffer (included in the Innuprep Kit (845-KS-20400250, Innuscreen GmbH, Germany) 10 mM Tris-HCl; 1 mM EDTA; pH 8.0). Lysozyme and lysis buffer were added per the InnuPrep Kit protocol for RNA isolation from Gram-negative bacteria. Subsequent steps were performed according to the manufacturer’s protocol. After the RNA concentration was assessed using a nanodrop spectrophotometer, DNase treatment was done according to the manufacturer’s guidelines (DNase I, RNase-free, EN0521, Thermo Scientific, US). The cDNA was synthesized using iScript cDNA Synthesis Kit (708891, Bio-Rad, US) according to the manufacturer’s instructions. Samples were subjected to the following thermal cycling program: Priming for 5 min at 25 °C, Reverse transcription for 20 min at 46 °C, RT inactivation for 1 min at 95 °C, Hold at 4 °C indefinitely. Primers specific for Ct 16S and 23S rRNA genes were used to assess bacterial load. The sequences (5’ -> 3′) were as previously described (Bellmann-Weiler et al., 2018; Blumer et al., 2011). For each primer the qPCR Master mix was prepared using SYBR Green Supermix kit (1725271, Bio-Rad, US) and the reaction was performed with the following PCR settings at the CFX Duet Real-Time PCR System: Denaturation at 95 °C for 3 min, annealing at 95 °C for 15 s, 60 °C for 60 s (40 x), melt curve analysis 60°-> 95 °C in 5 s.

Relative expression of CtE 16S and 23S rRNA was quantified by RT-qPCR using the comparative 2^–ΔΔC_T_ method (ΔC_T_ = C_T__target − C_T__GAPDH). GAPDH served as the endogenous control because its C_T_ did not vary across untreated, antibiotic-treated, and DHP/Alg-treated A2EN cells. The CtE (A2EN + CtE) group was used as the calibrator and assigned a value of 1. For visualization, we plotted fold reduction vs. CtE (FR = 1/2^–ΔΔCt) on a log10 years-axis. The uninfected A2EN condition was omitted from FR plots because its background signal yields substantial FR values that compress the scale. Statistical significance was assessed by one-way ANOVA with Dunnett’s multiple comparisons versus CtE (n = 3). Normalization to β-actin produced the same conclusions.

### 2.10 Statistics

One-way ANOVA was used for statistical analysis, with significance indicated as *p ≤ 0.05, **p ≤ 0.01, ***p ≤ 0.001, ****p ≤ 0.0001. Analyses were conducted using GraphPad Prism 10 software.

## 3 Results

### 3.1 DHP does not exhibit cytotoxic effects on A2EN cells

**FIGURE 3:**
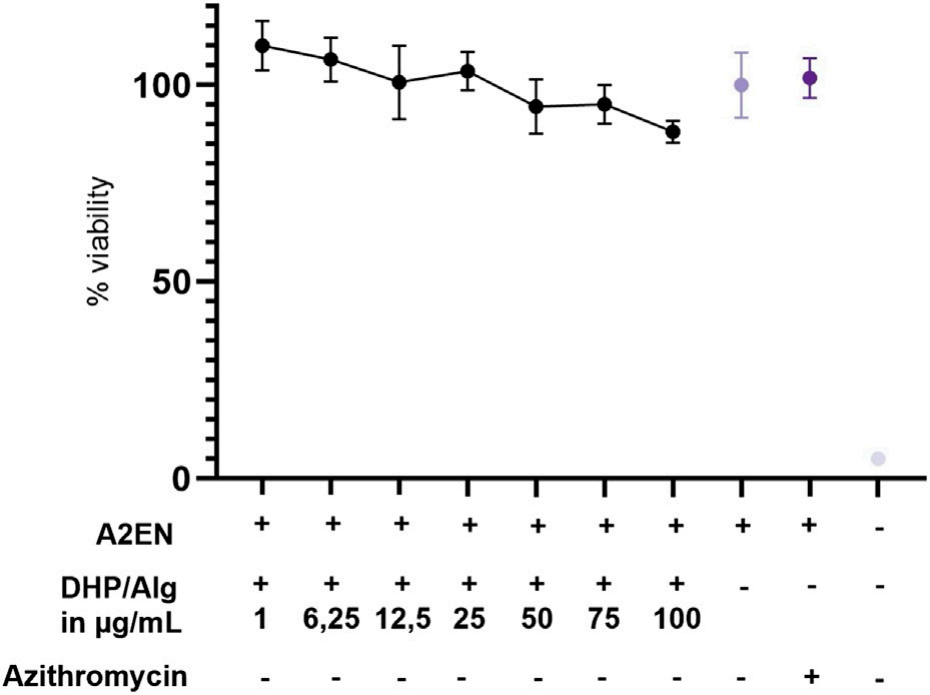
Cytotoxicity test of DHP/Alg treatment on A2EN cells. The DHP:Alg ratio is 1:2, and the DHP concentrations tested are shown on the x-axis on the graph (1–100 μg/mL). As controls only cells (A2EN), antibiotic treatment (A2EN + 1 μg/mL Azithromycin) and only medium were used. The y-axis shows cell viability as a percentage relative to untreated cells which were set to 100% viability. Error bars represent standard deviations from triplicates. Statistical test was done using a one-way ANOVA.

A cytotoxicity assay was performed to evaluate whether DHP/Alg treatment affects A2EN cell viability (Figure 3). The cells’ viability in the presence of DHP/Alg in concentrations tested (1–100 μg/mL with respect to DHP) was compared with the viability of untreated cells, which was set to 100% viable cells.

No significant reduction in viability was observed in the treatment of cells with DHP in the range of 1–100 μg/mL DHP/Alg. Treatment with 1–75 μg/mL DHP/Alg maintained viability close to 100%. A modest decrease in cell viability (<90%) was detected at 100 μg/mL, indicating limited cytotoxicity of DHP/Alg at this concentration. Therefore, lower concentrations (1–75 μg/mL) are preferable to minimize potential cytotoxic effects.

Further, before initiating infection experiments, we tested whether heat-inactivated CtE could account for cytotoxicity. By doing this, we wanted to ensure that any effects observed with live infection could be ascribed to viable *Chlamydia* rather than cellular debris. A2EN cells exposed to heat-inactivated showed no loss of viability, and viability remained unchanged when heat-inactivated CtE was combined with DHP/Alg (75–100 μg/mL) (CCK-8, 48 h; p > 0.05) (Supplementary Figure S1).

### 3.2 Comparison of DHP/Alg treatment strategies highlights simultaneous administration as most effective

As an initial prescreen, we used a flow cytometry-based infection assay to rank dosing/timing regimens before confirmatory culture work. We compared three DHP/Alg treatment strategies (details described in Section Material and Methods 2.6): 1) CtE pre-treated, 2) A2EN pre-treated cells, and 3) standard treatment (illustrated in Figure 1). Infection rates were determined by measuring CFSE-positive cells via flow cytometry, with the non-treated group set to 100% infection. The gating strategy and validation of cell viability by Live/Dead staining are shown in Supplementary Figure S2A–D, confirming that reduced CFSE positivity reflects decreased infection rather than cytotoxicity.

**FIGURE 4:**
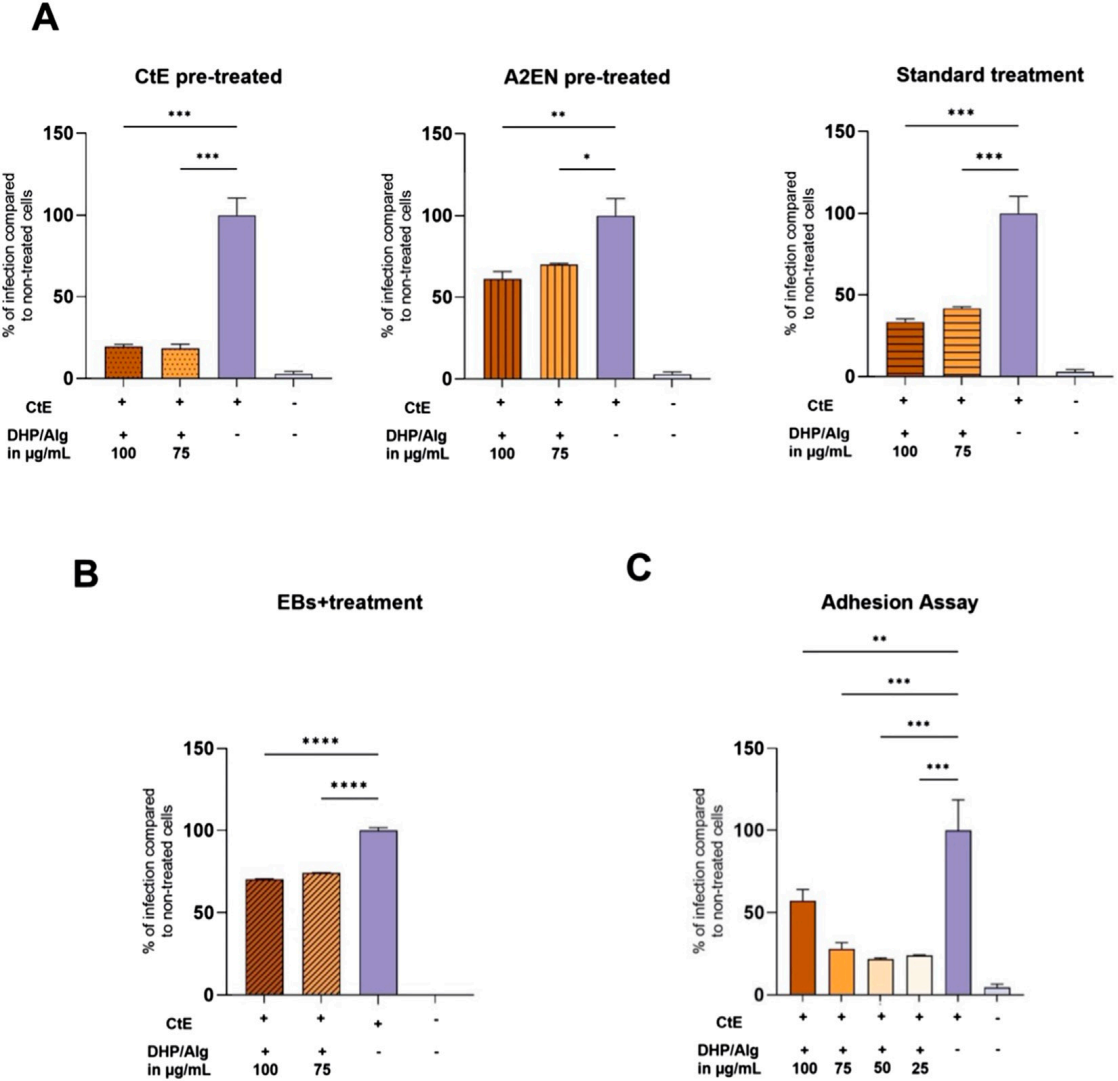
Infection levels measured in the infection and adhesion assays. Infection was conducted with CtE EBs (MOI = 10). The graphs display the percentage of infection relative to non-treated cells, set as 100%. **(A)** Infection assay. Three treatment conditions were evaluated: pre-treated EBs, pre-treated cells, and standard treatment. The level of infection was measured after 48 h. **(B)** Infection assay with an additional treatment option. Additionally, simultaneous treatment and infection were evaluated. The level of infection was measured after 48 h. **(C)** Adhesion assay. This graph illustrates the impact of DHP/Alg on the initial adhesion phase of CtE infection in host cells. Various concentrations of DHP/Alg (25–100 μg/mL) were tested. The percentage of infected cells (relative to the untreated control set to 100%) measured 1 h post-infection using flow cytometry is shown. A statistical test was done using a one-way ANOVA. The levels of statistical significance are indicated as *p ≤ 0.05, **p ≤ 0.01, ***p ≤ 0.001, ****p ≤ 0.0001.

As depicted in Figure 4A, pre-treated cells showed less infection reduction (for 100 μg/mL DHP/Alg, p ≤ 0.01; for 75 μg/mL DHP/Alg, p ≤ 0.05) compared to pre-treated EBs (p ≤ 0.001) and standard treatment (p ≤ 0.001). The most substantial reduction in infection could be observed when EBs were pre-treated with DHP/Alg. The standard treatment also showed a promising decrease in infection levels.

These results led us to add a fourth treatment strategy, which is the simultaneous administration of EBs and DHP/Alg treatment (Figure 1D). As shown in Figure 4B, simultaneous treatment resulted in a more substantial reduction (p ≤ 0.0001) in infection, suggesting a direct effect of DHP on the adhesion of EBs to the cells. Notably, this treatment group was also more homogeneous, with relatively small standard deviations between replicates.

### 3.3 Adhesion assay reveals anti-chlamydial activity of DHP/Alg

The adhesion assay via flow cytometry (Figure 4C) showed that with DHP/Alg treatment, internalization of EBs at 1-h post-infection is significantly reduced, in all tested concentrations (25–75 μg/mL) (p ≤ 0.001); (100 μg/mL) (p ≤ 0.01). Under these conditions, we did not detect a significant, dose-dependent change in adhesion; the slight shift at 100 μg/mL might be best explained by the modest viability decrease at that dose (Figure 3). Reduced viability at the time of measurement could alter the proportion of cells detected as positive, without reflecting an actual increase in adhesion. Overall, these data are most consistent with a direct effect on the pathogen and/or very early post-entry steps. Given the clinical relevance of targeting established infections, we further explored the effectiveness of standard DHP/Alg treatment.

### 3.4 Treatment with DHP/Alg reduces chlamydial infection *in vitro*

**FIGURE 5:**
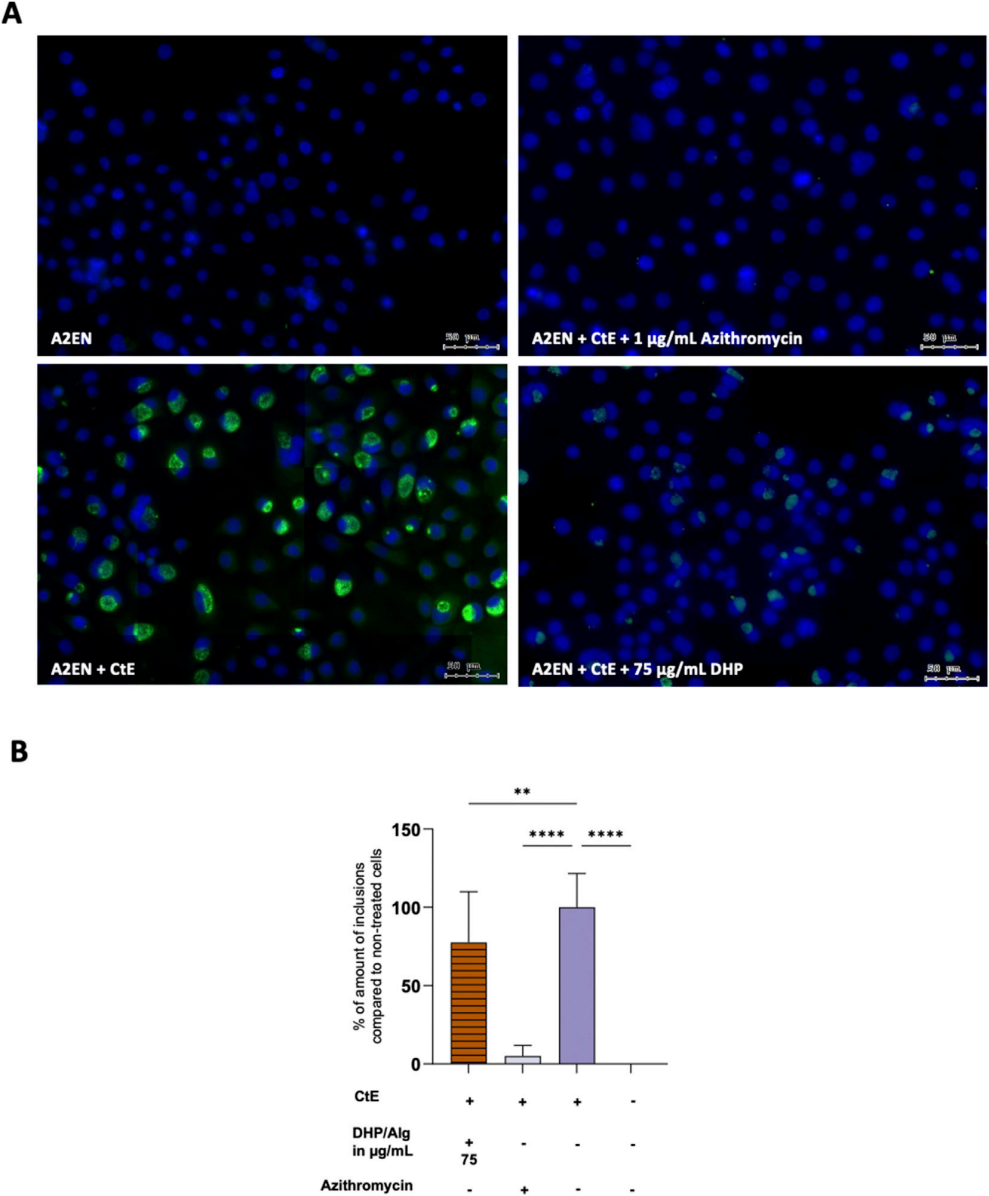
Infection assay via fluorescence microscopy. **(A)** Fluorescent images. A2EN (above panel, left), A2EN + CtE +1 μg/mL Azithromycin (above panel, right), A2EN + CtE (below panel, left), A2EN + CtE +75 μg/mL DHP/Alg (below panel, right). **(B)** Semiquantitative analysis of coverslips. The x-axis shows the different conditions while the y-axis shows the percentage of infection, with untreated cells set as the 100% infection baseline. A statistical test was done using a one-way ANOVA. The levels of statistical significance are indicated as *p ≤ 0.05, **p ≤ 0.01, ***p ≤ 0.001, ****p ≤ 0.0001.

Microscopy analysis of A2EN cells infected with CtE revealed large inclusions characteristic of CtE infection (Figure 5). Control samples: A2EN cells alone and A2EN cells treated with the antibiotic displayed no visible inclusions (as expected for uninfected cells). DHP/Alg treatment resulted in a significant reduction in the number of chlamydial inclusions. Semi-quantitative assessment further indicated that 75 μg/mL DHP/Alg had a strong anti-chlamydial effect (p ≤ 0.01).

**FIGURE 6:**
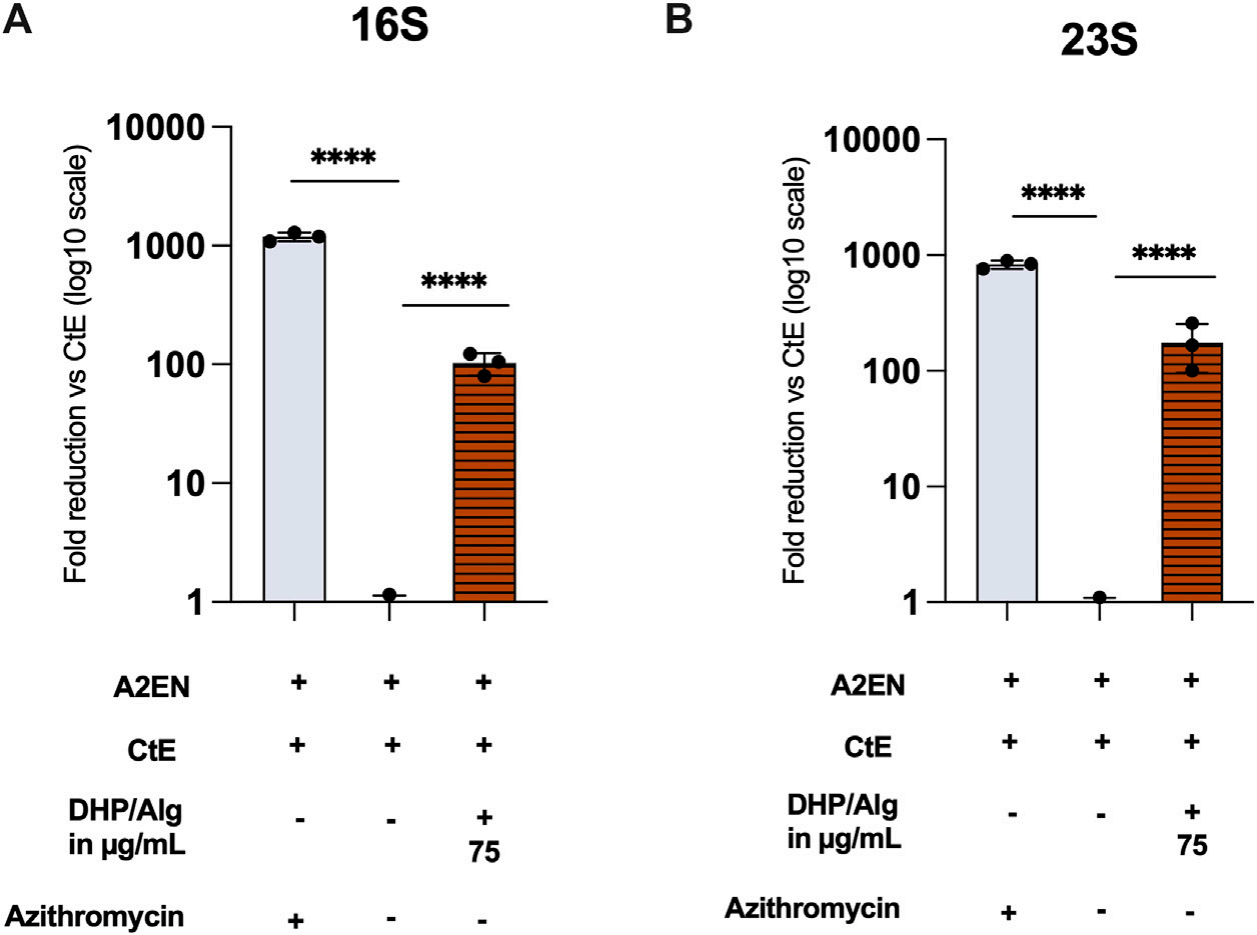
Infection assay via RT-qPCR. **(A)** 16S and **(B)** 23S CtE rRNA in A2EN cells, normalized to GAPDH and calculated by 2^–ΔΔCt (CtE = 1). Mean ± SD (n = 3) with Dunnett’s test vs. CtE (****p ≤ 0.0001). Plotted as fold reduction vs. CtE (FR = 1/2^–ΔΔCt) on a log10 scale; A2EN omitted due to background-level signal.

To corroborate these findings and quantify the bacterial burden more precisely, qPCR was performed to measure bacterial 16S and 23S rRNA expression (Figure 6). While the inhibitory effect of DHP/Alg was less pronounced compared to antibiotic treatment, exposure to 75 μg/mL DHP/Alg resulted in a highly significant reduction in both 16S and 23S rRNA levels (p ≤ 0.0001 for each) relative to CtE-infected untreated controls. This demonstrates that DHP/Alg effectively reduces the intracellular bacterial load, although not to the same extent as antibiotics. Also, qPCR detected residual 16S and 23S rRNA transcripts even in antibiotic-treated samples, although no chlamydial inclusions were visible by microscopy, which most likely represents traces of non-viable *Chlamydia*. Taken together, these results confirm the antibacterial activity of DHP/Alg and establish 75 μg/mL as the most effective concentration tested for suppressing CtE infection *in vitro*.

## 4 Discussion

Ct infections remain a significant public health concern. Despite extensive efforts by numerous research groups working on a vaccine (Poston et al., 2025; de la Maza et al., 2020; de la Maza et al., 2017; I et al., 2016; Inic-Kanada et al., 2015; Olsen et al., 2021; Hafner et al., 2014; Abraham et al., 2019; Kari et al., 2011), no approved vaccine is currently available. Antibiotics remain the primary treatment option and are generally effective, but treatment gaps persist due to antibiotic shortages and access issues, particularly in low- and middle-income countries. A 2022 survey of 29 European national pharmacist organizations revealed that 79% of respondents reported shortages of anti-infective agents, including antibiotics, in the past year (Key European Bodies Unite to Prevent Antibiotic Shortages, Medscape, 18 July 2023). These shortages can lead to incomplete or inappropriate treatment, such as overuse of broad-spectrum antibiotics and prolonged infections.

Despite efforts like the WHO’s AWaRe classification of antibiotics (AWaRe classification of antibiotics for evaluation and monitoring of use, 26 July 2023), access to antibiotics remains a significant concern. While *Chlamydia* has not yet shown widespread antibiotic resistance, the global rise of antimicrobial resistance in other bacterial infections highlights the importance of continued surveillance. Given these challenges, developing novel treatments, including both antimicrobial and immunotherapeutic strategies, remains crucial in the fight against Ct infections.

We investigated DHP/Alg as a potential novel therapeutic option for treating genital Ct infections. Our findings provide evidence of DHP/Alg’s therapeutic potential against Ct infections. Importantly, DHP/Alg treatment demonstrated no cytotoxic effect on A2EN cells in all tested concentrations, aligning with previous studies that confirmed the safety of similar formulations on ocular epithelial cells (Spasojević et al., 2016).

The A2EN cell line, an immortalized endocervical epithelial cell line, has been used as an *in vitro* model for studying genital Ct infections. This model is highly relevant for investigating sexually transmitted diseases, genital tract pathogenesis, and host immune response (Buckner et al., 2011). A2EN cells are a more physiologically relevant model for studying genital Ct infections than HeLa cells. While HeLa cells, a cervical adenocarcinoma-derived line, have been widely used in the field of chlamydial research, they possess significant limitations, including abnormal chromosome numbers and cancer-associated genetic mutations that can affect host-pathogen interactions (Lucey et al., 2009). In contrast, A2EN cells are derived from primary human endocervical epithelial cells and were immortalized using human telomerase reverse transcriptase (hTERT), maintaining key epithelial characteristics while avoiding the aberrations associated with cancer cell lines (Buckner et al., 2011). A2EN cells express relevant receptors and immune-related molecules, such as pattern recognition receptors (PRRs) and cytokines, which play crucial roles in the host response to chlamydial infection (Buckner et al., 2011). Additionally, A2EN cells produce mucus and exhibit tight junction formation, mimicking the physiological conditions of the endocervical epithelium more closely than HeLa cells, which lack these features (Edwards et al., 2022). Due to these properties, A2EN cells provide a superior *in vitro* model for investigating chlamydial pathogenesis, immune responses, and potential therapeutic interventions.

Lignin has been recognized for its antibacterial properties (Lobo et al., 2021), though its effects highly depend on extraction methods (Das et al., 2024; Ndaba et al., 2020). To address this variability and ensure reproducibility, we used a well-defined lignin model, DHP, which is particularly advantageous for biomedical applications. Our DHP formulation contained 15.7% LMW fractions (<3 kDa). Previous studies have shown that such LMW lignin fractions (0.2–3 kDa) in an Alg suspension promoted wound healing and showed an antimicrobial effect against *Staphylococcus* strains (Spasojević et al., 2024).

With the safety of the DHP/Alg treatment on A2EN cells ensured, we moved forward into investigating its anti-chlamydial properties, focusing on its potential to inhibit infection, disrupt bacterial adhesion, and reduce intracellular replication.

As an initial prescreen, we used a cost-effective flow cytometry-based infection assay (Schnitger et al., 2007) to rank dosing/timing regimens before confirmatory culture work. This assay showed that DHP/Alg significantly reduces chlamydial infection, with the most substantial effect when co-administered with CtE. By contrast, in the invasion assay we detected a significant overall reduction in adhesion with DHP/Alg, but this effect was not dose dependent; the slight shift at 100 μg/mL is best explained by the modest viability decrease at that dose. Together, these results underscore the importance of timing and duration: when free EBs are present, they are most likely to be directly affected by DHP, and infectivity is reduced; once intracellular, effects may accrue only after *de novo* EB release, implying that prolonged administration could be advantageous in practice.

Our results suggest that DHP might directly affect the EBs, disrupting their ability to adhere to host cells and interfering with the internalization process. As a lignin model, DHP may destabilize chlamydial membranes through non-specific mechanisms such as enzyme inhibition and metal ion complex disruption (Das et al., 2024). Similar antimicrobial mechanisms have been reported for olive oil polyphenols, which disrupt the structural integrity of chlamydial outer layers (Di Pietro et al., 2023). Investigating potential structural rearrangements in the chlamydial membrane using targeted staining approaches could provide further mechanistic insights.

Recognizing the importance of addressing established infections in clinically relevant scenarios, we focused on evaluating the “standard treatment” regimen with DHP/Alg. Fluorescence microscopy revealed a significant reduction in inclusion number, with the most pronounced effect at 75 μg/mL DHP/Alg. These findings were further confirmed by qPCR analyses. The anti-microbial effects of the DHP/Alg treatments are in line with previous reports demonstrating DHP’s efficacy against a range of Gram-positive and Gram-negative bacteria (Spasojević et al., 2016; Zmejkoski et al., 2018). In addition to these broad-spectrum antibacterial properties, dehydrogenated polymers of coniferyl alcohol, such as DHP, exhibit excellent radical stabilization capacity (Gerbin et al., 2020), which has been associated with vigorous antibacterial activity in various applications (Makwana et al., 2015). These intrinsic chemical features may further contribute to the observed anti-chlamydial effects in our study.

## 5 Conclusion

In this study, we evaluated the cytotoxicity and anti-chlamydial potential of DHP/Alg formulations in human A2EN epithelial cells. Our results demonstrate that DHP/Alg is well tolerated at concentrations up to 75 μg/mL, with only modest cytotoxicity observed at 100 μg/mL. Flow cytometry-based prescreen infection assays revealed that all treatment regimens reduced Ct infection, with simultaneous administration of DHP/Alg and EBs exerting the strongest and most consistent effect. Adhesion assay further confirmed that DHP/Alg interferes with early infection events, most likely by directly targeting EBs and/or initial host–pathogen interactions.

Taken together, our findings highlight DHP/Alg as a promising candidate for further development as an adjunct or alternative strategy to combat chlamydial infections. Future studies will include expanded *in vitro* analyses against both ocular and genital *Chlamydia* serovars to confirm the broader therapeutic potential of DHP/Alg and to elucidate its mechanism of action, as well as *in vivo* safety and efficacy testing in a guinea pig model of ocular chlamydial infection (Filipovic et al., 2017).

## Data Availability

The raw data supporting the conclusions of this article will be made available by the authors, without undue reservation.

## Abbreviations

DAPI, 4′,6-diamidino-2-phenylindole; CFSE, Carboxyfluorescein succinimidyl ester; Ct, *Chlamydia trachomatis*; CtE, Ct Serovar E; DHP, Dehydrogenation polymer of coniferyl alcohol; DHP/Alg, DHP in Alginate hydrogel; DPBS, Dulbecco’s Phosphate-Buffered Saline; EBs, Elementary bodies; KSFM, Keratinocyte Serum Free Medium; LMW, Low-molecular weight; MOI, Multiplicity of infection; PID, Pelvic inflammatory disease; RT, Room temperature; STI, Sexually transmitted infection.
