# Correction: Antibacterial effects of coniferyl alcohol-derived dehydrogenation polymer on chlamydial infection *in vitro*

**DOI:** 10.3389/fchem.2026.1781504

**Published:** 2026-01-20

**Authors:** Anna Pfundner, Tamara Weinmayer, Nora Geissler, Ana Kovacevic, Dragica Spasojevic, Ksenija Radotic, Marijana Stojanovic, Irma Schabussova, Ursula Wiedermann, Aleksandra Inic-Kanada

**Affiliations:** 1 Institute of Specific Prophylaxis and Tropical Medicine, Center for Pathophysiology, Infectiology and Immunology, Medical University of Vienna, Vienna, Austria; 2 Institute of Immunology, Virology, Vaccines and Sera – Torlak, Belgrade, Serbia; 3 Institute for Multidisciplinary Research, University of Belgrade, Belgrade, Serbia; 4 Institute for Biological Research “Siniša Stanković” - National Institute of Republic of Serbia, University of Belgrade, Belgrade, Serbia

**Keywords:** chlamydial infection, sexually transmitted infection, *Chlamydia trachomatis*, treatment, lignin, dehydrogenation polymer

The funder Ministry of Science, Technological Development and Innovation of the Republic of Serbia], grant number 451–03-66/2024–03/200053, to Dragica Spasojevic and Ksenija Radotic, was erroneously omitted. The correct **Funding** statement reads:

“This research was funded by the Austrian Science Fund (FWF) projects PAT5452224 to AIK (10.55776/PAT5452224), PAT6619324 to AIK (10.55776/PAT6619324), and P34867 to IS and by the OeAD projects RS 16/2024 to AIK and CZ 04/2024 to IS. The research was also supported by the Ministry of Science, Technological Development and Innovation of the Republic of Serbia by grant No. 451–03-66/2024–03/200053 to DS and KR. For open access purposes, the authors have applied a CC BY public copyright license to any author-accepted manuscript version arising from this submission.”

The original article has been updated.

