# Supplementary material for: Antibacterial effects of coniferyl alcohol-derived dehydrogenation polymer on chlamydial infection in vitro
Source: Front Chem. 2025 Oct 10;13:1654478. doi: 10.3389/fchem.2025.1654478 (PMC12550777; doi:10.3389/fchem.2025.1654478)
Supplement: Supplementary file 1 [file DataSheet1.pdf]

Supplement

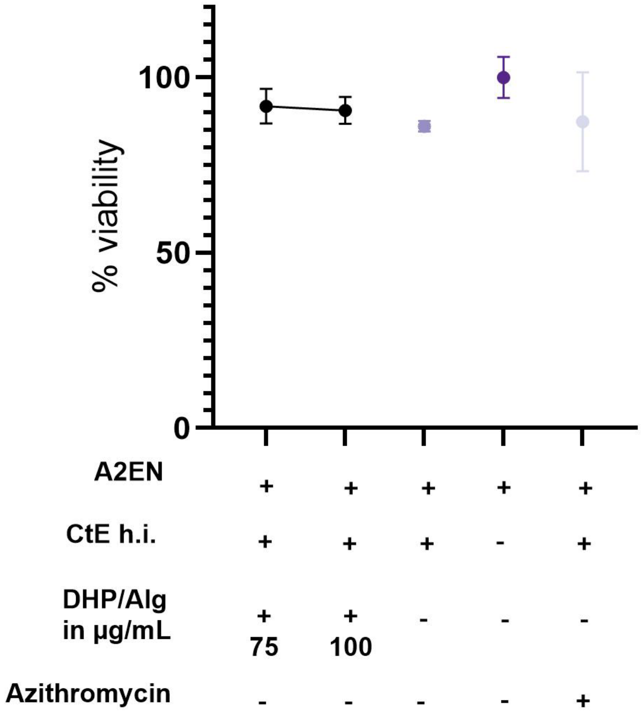

Supplementary Figure 1. Cytotoxicity test of heat-inactivated CtE in combination with DHP/Alg on A2EN cells after 48h. The DHP:Alg ratio is 1:2, and the DHP concentrations tested are shown on the x-axis on the graph (75, 100 µg/mL). As controls only cells (A2EN), cells with heat-inactivated CtE and cells with heat-inactivated CtE and antibiotic treatment (1 µg/mL Azithromycin) were used. The y-axis shows cell viability as a percentage relative to untreated cells without heat-inactivated CtE, which were set to 100% viability. Error bars represent standard deviations from triplicates. A statistical test was done using a one-way ANOVA.

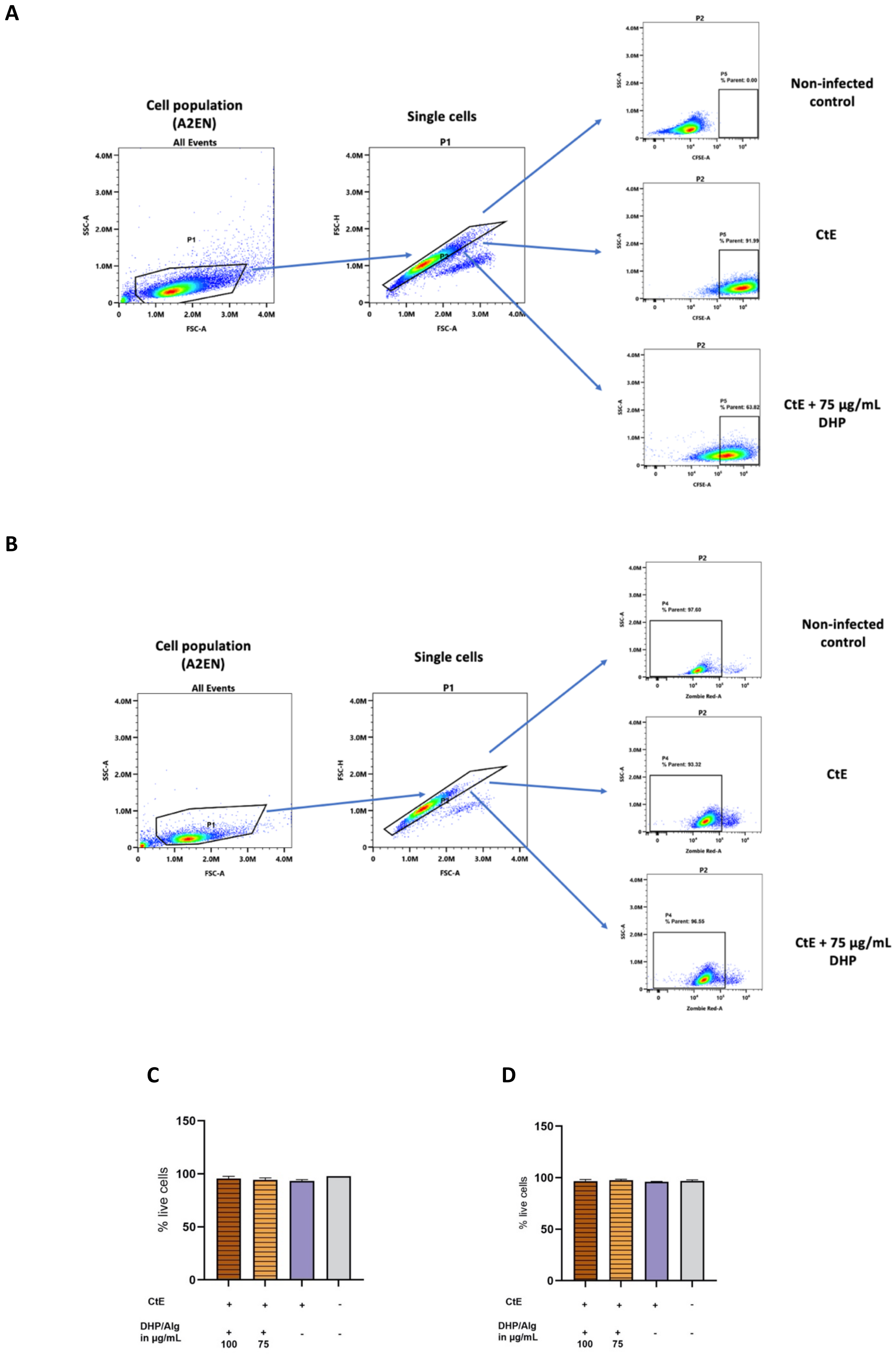
A
Cell population (A2EN)
All Events
Single cells
P1
P2
Non-infected control
CtE
CtE + 75 µg/mL DHP
SSC-A
FSC-A
FSC-H
CFSE-A
B
Cell population (A2EN)
All Events
Single cells
P1
P2
Non-infected control
CtE
CtE + 75 µg/mL DHP
Zombie Red-A
C
D
% live cells
CtE
DHP/Alg in µg/mL
100
75

Supplementary Figure 2. Flow cytometry gating strategy and Live/Dead staining. (A) Representative gating strategy for CFSE-based detection of CtE infection. Debris was excluded by FSC/SSC gating, singlets were selected by FSC-A vs. FSC-H gating, and infected cells were identified by CFSE fluorescence. This method served as a relative, cost-efficient pre-screening tool prior to confirmation by microscopy and qPCR. **(B)** Gating strategy for Live/Dead staining using the Zombie Red Fixable Viability Kit (BioLegend). **(C)** Representative Live/Dead staining of CtE-infected cells at MOI 10. **(D)** Live/Dead staining of CtE-infected cells at MOI 1. In both (C) and (D), cell viability remained consistently high (~90–100%) across treatments, confirming that reduced CFSE positivity reflected decreased infection rather than treatment-induced cytotoxicity.
